# MED33/447: Moving Medical Curriculum to the World Wide Web: Practical Tips On Using Macromedia Flash Animation in a Case Study on 'Reproductive Physiology'

**DOI:** 10.2196/jmir.1.suppl1.e70

**Published:** 1999-09-19

**Authors:** C Kesler, D Balch

**Affiliations:** ^1^Division of Continuing Studies: Global CampusEast Carolina UniversityGreenvilleUSA

**Keywords:** Multimedia, Flash Animation, Medical Education, Reproductive Physiology, Streaming Media, Online Curriculum

## Abstract

**Introduction:**

The internet combined with browser-based graphics, animation and streaming media offer a unique opportunity to reach rural students in both synchronous and asynchronous modes, while simultaneously augmenting the resources to students attending class in person.

**Methods:**

This presentation will showcase a semester long course in the undergraduate Nursing curriculum at East Carolina University School of Nursing. The three-hour weekly course utilized streaming media, graphics and animation to educate nursing students. Results:

The case study will highlight the production management that went into the development and the problems encountered and solved. Evaluations of both residential and remote students were compared showing little difference in retention.

**Discussion:**

Animations and project management will be broken down into their development components and processes will be explained.

